# Intra-arterial induction high-dose chemotherapy with cisplatin for oral and oropharyngeal cancer: long-term results

**DOI:** 10.1038/sj.bjc.6601674

**Published:** 2004-03-02

**Authors:** A F Kovács

**Affiliations:** 1 Department of Maxillofacial Plastic Surgery, Johann Wolfgang Goethe-University Medical School, Frankfurt am Main, Germany

**Keywords:** intra-arterial chemotherapy, head and neck neoplasms, oral cancer, cisplatin, neoadjuvant treatment

## Abstract

Intra-arterial (IA) chemotherapy for curative treatment of head and neck cancer experienced a revival in the last decade. Mainly, it was used in concurrent combination with radiation in organ-preserving settings. The modern method of transfemoral approach for catheterisation, superselective perfusion of the tumour-feeding vessel, and high-dose (150 mg m^−2^) administration of cisplatin with parallel systemic neutralisation with sodium thiosulphate (9 g m^−2^) made preoperative usage feasible. The present paper presents the results of a pilot study on a population of 52 patients with resectable stage 1–4 carcinomas of the oral cavity and the oropharynx, who were treated with one cycle of preoperative IA chemotherapy executed as mentioned above and radical surgery. There have been no interventional complications of IA chemotherapy, and acute side effects have been low. One tracheotomy had to be carried out due to swelling. The overall clinical local response has been 69%. There was no interference with surgery, which was carried out 3–4 weeks later. Pathological complete remission was assessed in 25%. The mean observation time was 3 years. A 3-year overall and disease-free survival was 82 and 69%, respectively, and at 5 years 77 and 59%, respectively. Survival results were compared to a treatment-dependent prognosis index for the same population. As a conclusion, it can be stated that IA high-dose chemotherapy with cisplatin and systemic neutralisation in a neoadjuvant setting should be considered a feasible, safe, and effective treatment modality for resectable oral and oropharyngeal cancer. The low toxicity of this local chemotherapy recommends usage especially in stage 1–2 patients. The potential of survival benefit as indicated by the comparison to the prognosis index should be controlled in a randomised study.

Intra-arterial (IA) local chemotherapy for curative treatment of head and neck cancer experienced a revival in the last decade. Mainly, it was used in concurrent combination with radiation (Robbins *et al*, 1994; Shibuya *et al*, 1994; von Scheel *et al*, 1996; Fuwa *et al*, 2000; Furutani *et al*, 2002; Yamashita *et al*, 2002; Kitagawa *et al*, 2003) or alone (Nakasato *et al*, 2000). The drugs used mainly have been cisplatin or carboplatin. Administration was either continuously over a temporal retrograde approach (Fuwa *et al*, 2000; Nakasato *et al*, 2000; Furutani *et al*, 2002) or by repeated bolus infusions over a transfemoral anterograde approach (Robbins *et al*, 1994). The temporal approach had the general disadvantage of not being selective or superselective. The main aim of these trials was organ preservation in patients with advanced cancer, and the reported overall survival rate in the studies with acceptable minimal observation time (median 3 years) were 39% (von Scheel *et al*, 1996), 64% (local control rate; Fuwa *et al*, 2000), and 58.9% (Furutani *et al*, 2002). The largest population consisting of 213 patients was the one Robbins *et al* (2000b) reported, and overall and cancer-related 5-year survival of these patients with stages 3–4 disease was 38.8 and 53.6%, respectively.

Truly neoadjuvant usage (before definitive treatment by surgery or radiation), with an exclusive transfemoral approach and superselective perfusion, has been rare (Korogi *et al*, 1995; Hirai *et al*, 1999; Benazzo *et al*, 2000; Damascelli *et al*, 2003). By far, the largest population (213 patients) in this group was reported by Kovács *et al* (1999) and Kovács (2003a); the other investigators having reported between 13 and 23 patients. Performing a modification of the method adopted by Robbins *et al* (1994, 2000b) for the head and neck (IA high-dose cisplatin plus systemic antagonisation with sodium thiosulphate), Kovács and co-workers could clinically and pharmacologically demonstrate a very low rate of acute side effects in the neoadjuvant setting (Kovács *et al*, 2002; Kovács, 2003a, 2003b; Tegeder *et al*, 2003). In the period between 1996 and today (September 2003), over 350 patients with primary and recurrent malignancies of the head and neck have been treated with IA high-dose cisplatin in the author's institution.

Nearly all hitherto reports concentrated on interventional safety and tumour response. The proceedings of an international meeting on IA chemotherapy in head and neck cancer at Hanover in 1997 demonstrated the problems of many studies concerning patient survival. For example, Japanese results could not easily be compared to European ones due to a high number of maxillary sinus carcinomas, regular temporal retrograde approach with continuous infusion, various combinations with radiation, which were frequently unplanned, and rarely used anticancer drugs like peplomycin (Hibi *et al*, 1999; Inuyama, 1999). Molinari, who had the hitherto greatest European experience (168 patients) with neoadjuvant IA chemotherapy, complained about the difficulties of providing homogeneous populations (Molinari *et al*, 1999). Diverse use of antineoplastic agents and mingling with postoperative surgery considerably reduced the number of patients in whom survival could be attributed to the additional effect of IA chemotherapy.

The aim of the presented paper is to discuss long-term survival of a population of 52 patients with resectable oral and oropharyngeal cancer solely treated with neoadjuvant IA high-dose chemotherapy and surgery with curative intention, and to show the feasibility, safety, and efficacy of that neoadjuvant treatment modality.

## MATERIAL AND METHODS

The demographic and pretherapeutic tumour-related data of the patients are summarised in Tables 1

**Table 1:** Patient characteristics

**Characteristic**	***n* (%)**
No. patients	52 (
	
*Gender*	
Male	28 (54)
Female	24 (46)
	
*Median age (years) (range)*	66 (38–89)
	
*ECOG performance status*	
0	26 (50)
1	23 (44)
2	2 (4)
3	1 (2)
	
*Primary tumour site*	
Floor of the mouth	17 (33)
Tongue	15 (29)
Mandibular alveolar process	7 (13)
Oral cheek mucosa	2 (4)
Retromolar trigone	3 (6)
Maxilla	1 (2)
Oropharynx	7(13)
	
*Clinical stage (UICC)*	
1	11 (21)
2	17 (33)
3	6 (11)
4	18 (35)

ECOG = Eastern Cooperative Oncology Group. Stage according to Union Internationale Contre le Cancer (UICC; Sobin and Wittekind, 1997).

and 2

**Table 2:** Distribution of cT and cN stages at baseline

	**cN0**	**cN1**	**cN2a**	**cN2b**	**cN2c**	**Total**
cT1	11	0	0	0	0	11 (21%)
cT2	17	3	0	0	2	22 (42%)
cT3	3	0	0	0	1	4 (8%)
cT4	5	4	0	4	2	15 (29%)
						
Total	36	7	0	4	5	52 (100%)

CT=clinical tumor classification. CN=clinical nodal classification.

. All tumours were histologically confirmed and previously untreated squamous cell carcinomas.

All patients of this study were considered fit for anaesthesia, and primaries and neck disease were judged to be resectable.

All oral cancer patients of our institution have been treated with a multimodality regimen consisting of neoadjuvant IA chemotherapy, surgery, and adjuvant radiation with concurrent systemic chemotherapy (Kovács, 2003b). Patients with T1 primaries were not treated with adjuvant radiotherapy. The omission of radiotherapy in the stage 2–4 patients reported in this paper was not the result of iatrogenic selection but of patient refusal (*n*=25), and objective contraindications for radiotherapy, like psychiatric disease (claustrophobia, organic brain syndrome, alcoholism with delirium; *n*=4), prolongated wound healing (*n*=7), dyspnoea (*n*=1), and earlier radiotherapy of the same area (*n*=4). The four patients with prior radiotherapy suffered from earlier primaries (each one from a non-Hodgkin lymphoma and oropharyngeal cancer, and twice from a laryngeal cancer), all more than 5 years before this study and without relapse. The male/female distribution showed less male patients than usual because men generally were more sympathetic to postoperative radiation. Finally, a population of 52 patients could be examined who were treated solely with neoadjuvant IA high-dose chemotherapy and surgery. Approval of the local ethical committee was given, and patients signed informed consent.

The routine pretherapeutic staging took place using palpation, ultrasound, CT, and MRT for diagnostics of neck lymph nodes as well as PET for diagnostics of secondary tumours, neck lymph node affection, and distant metastases.

Every patient was treated initially with one cycle of adjuvant IA high-dose chemotherapy.

In the morning of the intervention, the patients received 74 mg dolasetron and 75 mg prednisolone intravenously (i.v.) Afterwards, 1.5 l of a full electrolyte solution including 20 mval potassium chloride were infused i.v. over a period of 2 h. Then the right femoral artery was catheterised using a four-french catheter containing a coaxial microcatheter. After superselective visualisation of the tumour-feeding vessel using fluoroscopy and a contrast medium, 150 mg m^−2^ cisplatin (medac GmbH, Hamburg, Germany; maximal absolute dose 300 mg) dissolved in 500 ml 0.9% saline solution was infused at 2 ml s^−1^ with controlled pressure. For analgesia, 0.1–0.3 mg fentanyl was delivered i.v. (and on occasions of tooth aching 5–15 mg mepivacain) into the perfused artery. After 10 s, an i.v. infusion of 9 g m^−2^ sodium thiosulphate was administered in parallel to the perfusion. After chemoperfusion was completed, no more sodium thiosulphate was given. Starting in May 2000, tumour sites in the floor of mouth and the mobile tongue have been treated with an aqueous crystal suspension of cisplatin (5 mg in 1 ml); the absolute dose of cisplatin has been the same (Kovács *et al*, 2002). After the treatment, 1 l of full electrolyte solution containing 20 mval potassium chloride was infused again i.v. during a period of 5 h. The next day, the patients were hyperhydrated with 3 l of a two-third electrolyte solution, received thrombosis prophylaxis with heparin s.c., and dolasetron i.v. if nausea was present. Routine laboratory checks were made on alternate days. Ward stay lasted between 4 and 6 days for most patients. The side effects of the cycles according to WHO (Miller *et al*, 1981) were noted.

Remission was assessed clinically (by inspection and palpation) and by CT 3 weeks after the first cycle. The remission was graded as: CR=complete remission, a complete disappearance of local tumour mass; PR=partial remission, a reduction of local tumour mass of more than 50%; SD=stable disease, a reduction of local tumour mass of less than 50% or identical local tumour mass; PD=progressive disease, continued growth of the tumour.

The second stage was the operation. As a therapeutically effective ‘downstaging’ of the tumour was considered pointless, all resections were performed according to the pretherapeutic tumour extension plus healthy margins, as documented by pretherapeutic photography and CT.

Treatment of the neck was carried out according to the guidelines of the DÖSAK (Bier, 1982) with two important modifications: if pretherapeutic staging including PET resulted in the diagnosis of a clinically negative neck (cN0), only a suprahyoid neck dissection (SHND, a selective neck dissection that includes neck levels I and IIa) was carried out homolaterally, irrespective of the localisation and size of the primary tumour. In case of a positive pretherapeutic finding on whichever side of the neck, a Type III modified radical neck dissection (MRND III) was carried out. If the histologic examination of the neck specimen revealed positive nodes despite a pretherapeutic cN0 classification, an MRND of levels IIb, III, IV, and V was performed as early as possible. (The classifications of neck levels and types of operations follow the proposal of the American Head and Neck society (Robbins *et al*, 2000a).) Beginning in March 2000, sentinel node biopsy was executed instead of SHND in case of cN0 (Kovács *et al*, 2001). In case of positive sentinel nodes, an MRND was performed 1 week after SNB.

Survival of this population could be compared to a prognosis index. The treatment-dependent prognosis index (TPI) (Platz *et al*, 1985; Platz *et al*, 1992; Howaldt, 1999) was assessed for the population as demonstrated in Table 1. Treatment-dependent prognosis index combines data on tumour size (40 mm>*x*>40 mm), depth of tumour infiltration (5 mm>*x*>5 mm), evidence of fixed lymph nodes (yes/no), presence of distant metastases (yes/no), and age (50>50–70>70 years) based on two observational (one retrospective and one prospective) studies of the DÖSAK (Deutsch-Österreichisch-Schweizerischer Arbeitskreis für Tumoren im Kiefer-und Gesichtsbereich, German–Austrian–Swiss Cooperative Group on tumours of the maxillofacial region) comprising a total of 2500 patients. In all, 48 (2 × 2 × 2 × 2 × 3) combinations of these data are possible (=TPI groups 1–48). In every TPI group, patients are divided into two prognostic subgroups, according to the kind and success of treatment: those operated on radically and without/with clinical evidence of disease after completion of therapy, and those treated either surgically but not radically or with primary radiotherapy, and without/with clinical evidence of disease after completion of therapy. Each subgroup has its own prognostic prediction (expected survival at five points=years 1, 2, 3, 4, and 5) according to the results of the above-mentioned observational studies. For instance, the TPI 2 has a 5-year survival expectancy of 72% and the TPI 38 one of 46% in case of radical surgery leaving no clinical evidence of disease. Calculation of the index was carried out using the Cox regression. This enabled a control for the present population. The prognostic indices for patients operated on radically and without clinical evidence of disease could be used for the patients of the present study and resulted in expected survival rates as shown in Table 3

**Table 3:** Distribution of TPI with prognosis for patients operated on radically and without clinical evidence of disease

**TPI**	**Expected 3-year survival (%)**	**Expected 5-year survival (%)**	**Number of patients**
1	83	79	3
2	77	72	7
3	70	64	4
13	72	66	4
14	64	57	16
15	54	47	11
21	33	25	1
38	54	46	2
39	43	35	3
44	33	25	1

TPI=treatment-dependent prognosis index.

.

Cumulative survival and disease-free survival for all patients were calculated by the Kaplan–Meier method (Kaplan and Meier, 1958). The mean survival time including its 95% confidence was estimated (SPSS Statistical Algorithms, 1991), and the cumulative percentage of surviving subjects was calculated for 1, 2, 3, 4, and 5 years including 95% confidence intervals. The number of subjects at risk and cumulative events over time were calculated and are below the time axis of each Kaplan–Meier curve.

The difference of the TPI estimator with respect to the confidence range of the calculated survival was evaluated. The TPI estimator is predicted by a Cox regression equation based on the above-mentioned studies of the DÖSAK, while the estimators of the presented sample are based on the Kaplan–Meier method. Therefore, a classical test of difference (e.g. log rank) between the observed sample and population data could not be carried out. An indicator for any difference between the resulting curves for survival and expected survival could be that the TPI estimator is outside the confidence range for survival calculated in this sample.

## RESULTS

Intra-arterial high-dose chemotherapy was executed in every patient, once in 48 patients and twice in four patients (=56 interventions). In these cases of PR after the first cycle, assessed visually and by CT, the cycle was repeated, and these four patients (8%) have not been excluded because the study goals of feasibility, safety, and efficacy were not affected, and survival analysis was only potentially affected.

There have been no interventional complications. One adipose patient with a T2 cancer of the anterior floor of mouth developed a submental swelling with dyspnoea; a tracheotomy was executed 1 day after the intervention. The patient could be decannulated 3 days later without further complications. The acute side effects of IA chemotherapy are noted in Table 4

**Table 4:** Acute side effects of IA chemotherapy

**Toxicity**	**Grade I**	**Grade II**
Nausea (*n*)	6	2
Hypokalaemia (*n*)	11	0
Sideroemia (*n*)	9	0
Hyperglycaemia (*n*)	12	0
Hyperuremia (*n*)	2	0
Serum creatinine (*n*)	9	0
Hepatic enzymes (*n*)	6	0
Pain (*n*)	18	2
Leucocytosis (*n*)	15	0
Swelling (*n*)	7	0

Grades according to WHO (Miller *et al*, 1981).

IA=intra-arterial.

. In sum, IA chemotherapy had few and low-grade acute side effects.

Remissions as assessed clinically after the first cycle were: CR 20 patients (38%), PR 16 patients (31%), and SD 16 patients (31%). This indicates a clinical response rate (CR+PR) of 69%.

Surgery was carried out according to the generally accepted rules of radicality and reconstruction without unusual complications. Especially, microsurgery was not disturbed by alterations of the vessels used for chemoperfusion. The treatment of the neck is listed in Table 5

**Table 5:** Neck dissections

**Neck dissection**	**Number**
None	4
Ipsilateral MRND	8
Ipsilateral MRND, contralateral SHND	10
Ipsilateral SHND	5
Bilateral SHND	8
SNB	17

MRND=type III modified radical ND; SHND=suprahyoid ND; SNB=sentinel node biopsy.

. Four patients already had neck surgery due to earlier primaries at different sites. In sum, neck treatment had to be considered as conservative with the aim of preservation of functions. Histopathological examination of the primary specimen confirmed the ‘downstaging’ of the primaries, including a rate of 25% pathological CRs (Table 6

**Table 6:** Distribution of pT and pN stages post-treatment

	**pN0**	**pN1**	**pN2a**	**pN2b**	**pN2c**	**Total**
pT0	11	1	1	0	0	13 (25%)
pT1	19	4	0	0	0	23 (44%)
pT2	5	1	0	0	1	7 (13%)
pT3	0	0	0	0	0	0
pT4	4	3	0	2	0	9 (18%)
Total	39	9	1	2	1	52 (100%)

‘Downstaging’ of primaries following IA chemotherapy; cf. Table 2.

pT=pathological tumor classification. pN=pathological nodal classification.

). Pathological stages were: stage 0/11 patients, 1/19, 2/5, 3/6, and 4/11. Although local radicality was not affected by ‘downstaging’, surgery was facilitated by higher topographical clarity and a higher consistency of the tumours making them better palpable.

In all, 10 patients suffered from relapse: seven from local relapse and four from regional relapse in the neck. Five of these recurrences could be treated by salvage surgery.

**Figure 1:**
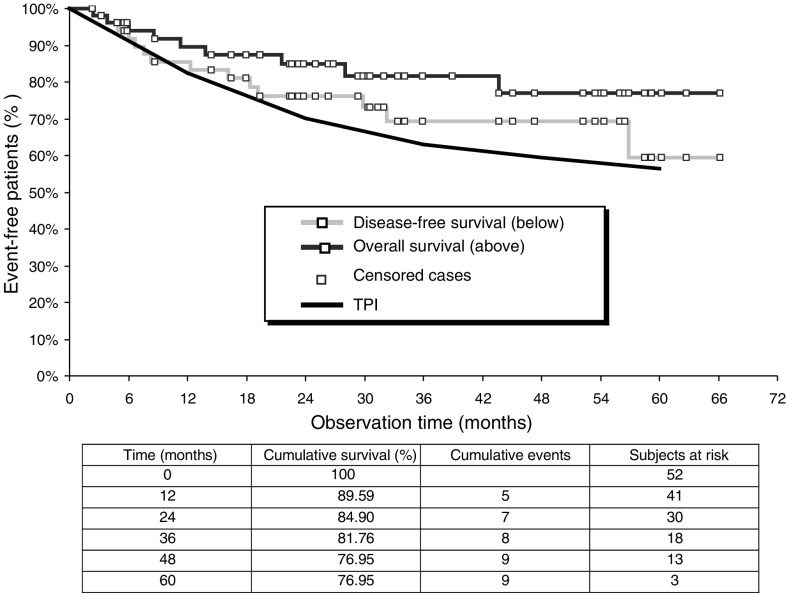
Overall and disease-free survival of 52 patients treated with neoadjuvant IA high-dose chemotherapy with cisplatin and radical surgery. Comparison with expected survival according to a treatment-dependent prognosis index TPI.

Patients were followed up for a mean period of 36 months (SD 18.9; range 5–67 months). Overall and disease-free survival is shown in Figure 1

. For survival, there have been 43 (83%) censored cases with a mean survival time of 55 months (95% confidence interval of 49–62 months). The mean disease-free survival time has been 49 months (95% confidence interval of 42–57 months). At 3 years, overall survival was 82% and disease-free survival was 69%. Cumulative survival rates at 5 years were 77 and 59%, respectively. Confidence intervals for survival are demonstrated in Table 7

**Table 7:** Cumulative percentage of surviving subjects including 95% confidence intervals

**Time (months)**	**Cumulative survival (%)**	**Lower 95% confidence**	**Upper 95% confidence**
12	90	81	98
24	85	75	95
36	82	70	93
48	77	63	91
60	77	63	91

. The survival expectancy for the whole population according to the TPI was 63% at 3 years and 56% at 5 years (Figure 1). The TPI estimator was at any point (1, 2, 3, 4, and 5 years) below the confidence range for survival (cf. Table 7).

## DISCUSSION

The randomised studies on neoadjuvant IA chemotherapy, conducted in the 80s of the last century, demonstrated a survival benefit for oral cancer patients. However, oral cancer patients have been a subset of larger study populations and, therefore, calculation could not be estimated appropriately. Arcangeli *et al* (1983) treated 72 head and neck cancer patients (stages 2–4) with retrograde continuous IA local perfusion with methotrexate (3–5 mg day^−1^, up to an overall dose of 90–120 mg) before definitive radiotherapy. Randomisation was against radiotherapy alone, and the complete study population comprised 142 patients. The subgroup of oral cancer patients had a 5-year survival of 54%, which was significantly better than the comparison (27%). An EORTC study (Richard *et al*, 1991) comprised 222 patients with resectable floor of mouth and oropharyngeal carcinomas. In all, 112 patients were administered retrograde IA vincristin (1 mg day^−1^) on 3 days and bleomycin (15 mg day^−1^) over 12 days preoperatively. Only oropharyngeal cancer patients underwent obligatory postoperative radiation, while radiation of floor of mouth cancer patients was dependent on surgical margins and lymph node state. Randomisation was against operation alone. The subgroup of floor of mouth cancer patients had a survival advantage, but oropharyngeal cancer patients did not. The third randomised study (Szabó *et al*, 1999) reported on 131 patients with sublingual and lingual carcinomas (stages T2–4NXMX). The drugs used were cisplatin (50 mg day^−1^) and epirubicin (60 mg day^−1^) given alternating to 47 patients over a period of 12 days; the approach was retrograde. Randomisation was against preoperative radiation. The 5-year survival of the patients with IA chemotherapy was not significantly better (38% *vs* 31%). The authors nevertheless stressed the higher quality of life after IA chemotherapy as compared to radiotherapy. Wound healing after surgery was not disturbed, and chronic side effects of radiation like xerostomia were lacking.

The report of Molinari *et al* (1999) made it unmistakably clear as to why this treatment procedure was abandoned: the problems and acute side effects of IA chemotherapy over a temporal approach were too severe. Cannulation attempts (11%) have been not successful; further complications have been dislocations and occlusions of catheters (8%), local inflammations (15%), neurological complaints like temporary paralysis of the facial nerve (4%), and discontinuations of perfusion (20%). Acute hematological side effects with the drugs methotrexate, bleomycin, adriamycin, and cisplatin have been observed in 2.8%.

It is obvious from the presented results that modern IA chemotherapy over a transfemoral approach had less interventional complications, had higher selectivity (no facial nerve damage), and transported higher doses to the tumour area (maximum dose 300 mg cisplatin) than possible with earlier methods due to systemic neutralisation.

The absolute survival results are better than reported elsewhere with other IA regimens (von Scheel *et al*, 1996; Robbins *et al*, 2000b; Furutani *et al*, 2002), and papers on neoadjuvant usage of IA chemotherapy failed to report long-term survival. The results of the present paper prove the feasibility, safety, and effectivity of neoadjuvant treatment with IA chemotherapy, and suggest that survival of patients treated with neoadjuvant IA chemotherapy and surgery was better than the survival expectancy calculated with the help of a prognosis index. Considering the fact that IA chemotherapy mainly has a local effect due to pharmacokinetics (Tegeder *et al*, 2003), neoadjuvant usage in patients with the small tumour stages 1 and 2 who had no clinical evidence of nodal disease would be most logical.

Volling *et al* (1994, 1999) conducted a prospective randomised trial on primary systemic chemotherapy with cisplatin and 5-fluorouracil. This internationally less known trial could demonstrate that especially patients with smaller resectable carcinomas of the tonsils and the floor of mouth benefited from this neoadjuvant treatment. The results of the present study point in the same direction. The acute side effects of a systemic chemotherapy, however, are much more frequent and higher. The extremely low acute side effects of IA high-dose chemotherapy with systemic neutralisation (Kovács, 2003b) strongly recommend this method when carried out over a transfemoral approach.

Although it could be demonstrated that complete and PRs of oral carcinomas have a prognostic value after local chemotherapy (Kovács 2003a), it would be too early to conclude a change in treatment for patients with local response. Therefore, surgical radicality has been maintained. Repetitions of courses did not alter the direction of initial response (Kovács, 2003b), which is the reason why one neoadjuvant course is adopted. In any way, it is possible to investigate the necessity of postoperative treatment modalities in patients with positive response, and to examine genetical changes in tumours following chemotherapy.

It is generally accepted that specific therapeutic recommendations can be expressed only based on results of prospective randomised studies. The statistical designs of such studies, however, were often criticised. The main problems, as in the studies mentioned above, were the high number of patients required in both study arms to produce hard results, and calculation of subsets in such heterogenous populations, which has to be considered as inappropriate. In the light of this problem, the consideration of a prognosis index, which is statistically more than a historical control, may be indicated as a control for a pilot study.

The TPI may be a better prognostic tool than TNM because it additionally comprises tumour infiltration depth and the age of the patients, which are accepted as highly relevant prognostic factors. It is based on a historical (retrospective and prospective) observation, but the indices were calculated using multivariate Cox's regression. The author could demonstrate in a monography on multimodality treatment of oral and oropharyngeal cancer that it is very difficult to surpass the survival expectancy based on the TPI (Kovács, 2003b). Therefore, the results presented in this paper can cautiously be interpreted as a survival benefit for the sample, and IA high-dose chemotherapy with cisplatin with systemic neutralisation with sodium thiosulphate can surely be used in oral and oropharyngeal cancer patients with great feasibility, safety, and effectivity. Low-grade toxicity would especially suggest usage for stage 1–2 patients. Proof of a survival benefit, however, requires a prospective randomised study.
